# The effect of Tai Chi practice on immunological function in cancer survivors: A protocol for systematic review

**DOI:** 10.1097/MD.0000000000021869

**Published:** 2020-09-04

**Authors:** Xuejiao Wang, Lei Xu, Ning Dai, Xingzhe Yang, Qingyun He, Libo Tan, Ruochong Wang, Feng Li

**Affiliations:** a Beijing University of Chinese Medicine; b Department of Liver Diseases, Guang’anmen Hospital, China Academy of Chinese Medical Sciences, Beijing, China.

**Keywords:** a systematic review, immunology, meta-analysis, oncology, protocol, Tai Chi

## Abstract

**Background:**

Tai Chi has been reported to be potentially effective for health and well-being of cancer survivors. It is worth to assess the effectiveness and safety of Tai Chi on immunological function in people with cancer.

**Methods:**

All relevant randomized controlled trials (RCT) will be reviewed on Tai Chi for immunological function in cancer survivors. Literature searching will be conducted until March 9, 2019 from major English and Chinese databases: Cochrane Library, Excerpta Medica Database (EMBASE), PubMed, CINAHL, Sprotdicus, American Association for Cancer Research Journals, Sino-Med database, China National Knowledge Infrastructure, Chinese Science and Technique Journals Database, and Wanfang Data Chinese database. Two authors will conduct data selection and extraction independently. Quality assessment will be conducted using the risk of bias tool recommended by the Cochrane Collaboration. We will conduct data analysis using Cochrane's RevMan software (V.5.3). Forest plots and summary of findings tables will illustrate the results from a meta-analysis if sufficient studies with the same outcomes are identified. Funnel plots will be developed to evaluate reporting bias.

**Results:**

This review will summarize the evidence on Tai Chi for immunological function in cancer survivors.

**Conclusions:**

We hope that the results of this study will provide significant evidence to assess the value Tai Chi practice on immunological function in cancer survivors.

**Ethics and dissemination:**

Ethics approval is not required as this study will not involve patients. The results of this study will be submitted to a peer-reviewed journal for publication.

## 1 Introduction

Cancer is a leading cause of global mortality and is responsible for 9.6 million deaths in 2018,^[1]^ and there will be >11 million cancer deaths worldwide in 2030,^[2]^ according to the World Health Organization (WHO). In some studies, the link between the immune system and cancer has been increasingly recognized. In tumor immunity, tumor cells act as antigens, immune cells and leukocytes infiltrate the tumor tissue function through chemotaxis to form immune defense.^[3]^ However, malignant tumors can change the host immune system to form mechanisms to evade immune system surveillance as they grow, and cancer patients’ immune system responses may be compromised.^[4–6]^ The past decade has witnessed a revolution in cancer treatments, shifting from widely targeted tumor drugs (e.g., chemotherapy and radiotherapy) to the use of antibody-based immunotherapy to regulate the immune response against tumors.^[7]^ Although with the advances of medical technology, overall cancer death rates continue to decline globally,^[8]^ cancer survivors are still suffering from adverse effects, such as reduced immune function,^[9–11]^ brought about by cancer and treatments for cancer. Immune function plays an important role in the prevention, progression, treatment, recurrence, and prognosis of cancer.^[12–17]^ Therefore, there is a need for safe, less invasive, and effective alternatives to improve the immune system.

So far, American College of Sports Medicine has hold two International Multidisciplinary Roundtable on Physical Activity and Cancer Prevention and Control aimed at putting forward high lever of recommendations for cancer survivors.^[18,19]^ What's more, Australia^[20]^ also put up their statement about exercise medicine in cancer management, in which the role of Tai Chi emerged. Indeed, Tai Chi, as an ancient Chinese mind-body fitness regimen, is guided to be mindful of their postures, movements, and breathing, with an intensive inwardly directed focus.^[21]^ To data, there has been some scientific or empirical evidences to support the claim that, Tai Chi can benefit immunological function in cancer survivors. For the body, Tai Chi as a form of moderate intensity aerobic exercise,^[22]^ has immunomodulatory effects that could alter multiple critical phases of immune system tumor cross-talk in both tumor initiation and progression, although this area of investigation clearly remains in its infancy. In view of mind-body intervention, Tai Chi might able to reverse the effects of acute and chronic stress and reduce the activation of sympathetic nervous system,^[23]^ focusing on aspects of immunity that are regulated by stress response mechanisms, namely inflammation and anti-viral related immune responses, etc.^[24]^

Up to now, there are nearly 20 systematic reviews about the Effect of Tai Chi practice on cancer related symptoms. The primary outcome measures of the reviews included short and long-term cancer-related fatigue, quality of life, aerobic capacity, muscular strength, and flexibility.^[25–39]^ Although adjusting the immune system can offer potential benefits to cancer survivors, there is no systematic review evaluating the effect of Tai Chi on immune system in cancer patients thus far. Khosravi et al^[40]^ conducted separate meta-analyses in cancer survivors to determine the effects of exercise training on pro- and anti-inflammatory markers, and immune cell proportions and function. But they just explored the cytokines which is included in >3 studies, and they combined Tai Chi and yoga together leading a mixed result; Koelwyn et al^[41]^ have made a very detailed review about the relationship between physical activity and immune surveillance, immune escape and immunogenic response to the tumor. Nevertheless, there rarely has introduction of Tai Chi. So the aim of this systematic review and meta-analysis of randomized controlled trials (RCT) is to evaluate current evidence and estimate the pooled effects of Tai Chi on the immune system, as well as adverse events.

## 2 Methods and analysis

### 2.1 Standards

The protocol of the meta-analysis will be developed according to the Preferred Reporting Items for Preferred Reporting Items for Systematic Reviews and Meta-Analyses protocols (PRISMA-P) guidelines. PRISMA-P is supplied in PRISMA checklist.^[42]^

### 2.2 Registration

Our meta-analysis protocol has been registered at the https://inplasy.com/ with registration number: INPLASY202060042. However, we plan to make a little change in the review in adjusting databases: deleting the Allied and Complementary Medicine Database (AMED) and inserting Chinese Science and Technique Journals Database (VIP) and Wanfang Data Chinese database. What's more, we would like to add 3 outcomes: cancer related fatigue (CRF), patient satisfaction, and adverse event.

### 2.3 Inclusion criteria

#### 2.3.1 Types of studies

RCTs assessing the effects of Tai Chi on immunological function and CRF in cancer survivors will be included. Reviews, systematic reviews, case reports, editorials, and study protocols will be excluded.

#### 2.3.2 Types of participants

This review will include cancer survivors diagnosed by applicable diagnostic criteria including European Society for Medical Oncology (ESMO) 2014; The World Health Organization 2017, etc, regardless of age, sex, tumor site, tumor type, tumor stage, and type of anticancer treatment received.

#### 2.3.3 Types of interventions

Studies that use any form of Tai Chi regardless of the form (e.g., 24-form, 54-form, 83-form Tai Chi) in any styles (e.g., Chen, Yang, Wu, and Sun) with a minimum frequency of once per week. The intervention could be provided in the format of a Tai Chi programme alone or as a Tai Chi programme in addition to another intervention. However, Tai chi softball, Tai chi fan exercise, and other exercises requiring the help of tools are excluded.

#### 2.3.4 Type of control

Studies with no treatment, other treatment forms like usual medical care, health education, pharmacotherapy, psychological therapy, cognitive behavioral therapy, and exercises other than Tai Chi such as walking, stretching, yoga, and dancing will be eligible.

#### 2.3.5 Types of outcome measures

All biomarkers related to the immune system including immune related cytokines and immune cells will be recorded including but not limited to pro-inflammatory cytokines IL-6, TNF (tumor necrosis factor)-α, C-reactive protein (CRP), anti-inflammatory cytokines IL-4, IFN (interferon)-γ, cortisol, and so on.

The score of cancer related fatigue.

The patient satisfaction and adverse event are to be measured.

### 2.4 Information sources

The following databases will be searched until March 9, 2019: Cochrane Library, Excerpta Medica Database (EMBASE), PubMed, CINAHL, Sprotdicus, American Association for Cancer Research Journals, Sino-Med database, China National Knowledge Infrastructure (CNKI), Chinese Science and Technique Journals Database (VIP), and Wanfang Data Chinese database.

### 2.5 Search strategies

All relevant published RCTs will be identified regardless of language or publication status. The English search terms will include “Tai Chi,” “Tai Chi Chuan,” “Tai ji,” “Tai-ji,” “Tai Ji Quan,” “taijiquan,” “neoplasms,” “cancer,” “carcino,” “tumor,” “randomized controlled trial,” “randomised controlled trial,” “controlled clinical trial,” “randomly,” “Clinical,” “trial,” “random,” “randomised,” and “randomized.” The Chinese searching terms will include Tai Chi (“*Tai_ji,”* “*Tai_ji_yun_shou,”* “*Tai_ji_cao,*” or “*Tai_ji_chuan*”), cancer (“*ai,”* “*ai_zheng,”* “*ai_zhong,*” or “*zhong_liu*”) and randomized (“*sui_ji,”* “*dui_zhao*”).

Detailed search strategies are available for example in Table 1: preliminary search strategy, appendixed below the references.

**Table 1:**
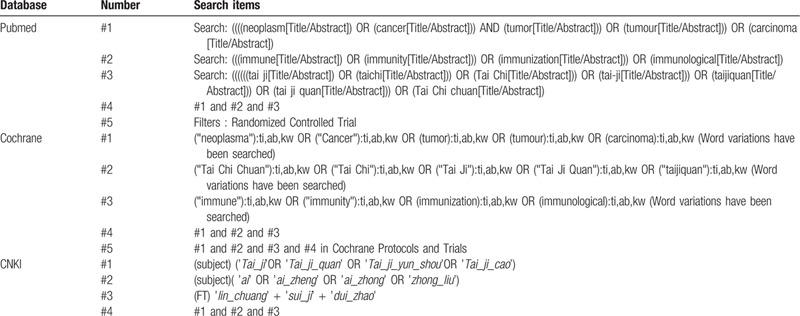
Search strategies.

### 2.6 Data selection and extraction

#### 2.6.1 Selection of studies

The search results from different databases will be imported into Endnote X8 (Thomson Research Soft, USA) and duplicate citations will be removed. Three authors (XW, QH, LX) will screen the titles and abstracts independently. Full texts of all potentially relevant studies will be retrieved. Any disagreement about the selection of studies will be resolved by discussion, and a third author (ND) will arbitrate when necessary.

**Figure 1:**
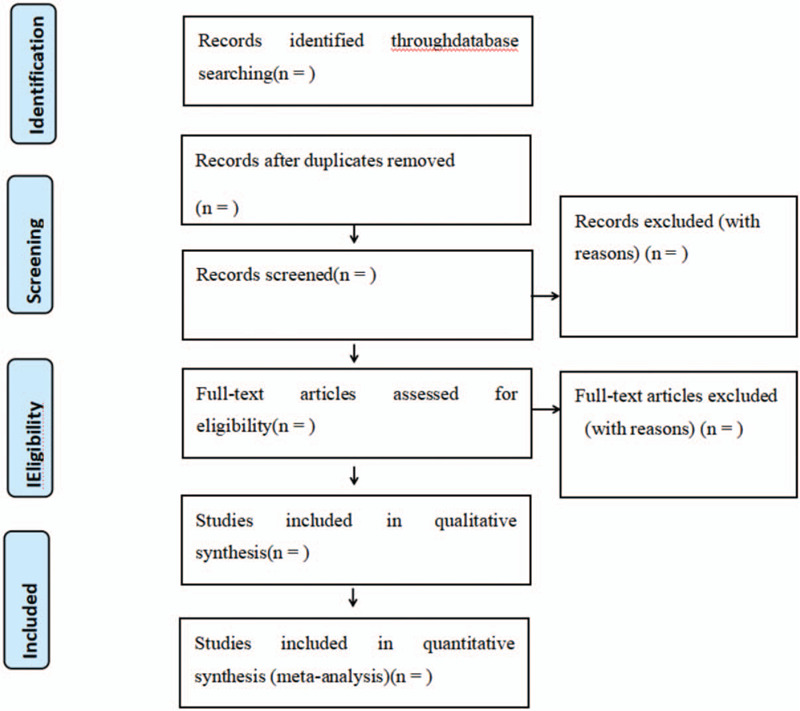
PRISMA flow diagram. PRISMA = Preferred Reporting Items for Systematic Reviews and Meta Analysis.

The selection procedure is shown in a Preferred Reporting Items for Systematic Reviews and Meta-Analyses flowchart in Fig. 1: Preferred Reporting Items for Systematic Reviews and Meta-Analyses (PRISMA) flow diagram, appendixed below the references.

#### 2.6.2 Data extraction and management

Three authors (XW, QH, and LX) will extract the data from the eligible studies independently by using a pre-designed form. Any disagreements will be resolved by discussion with a fourth author (ND). The extracted data will include the following information:

(1) publication information: authors, the country where the study was conducted, journal, title, and year of publication.
(2) study designs: multiple/single centre(s), parallel/cross-over, etc.
(3) participants: sample size, diagnostic criteria, characteristics of participants (e.g., age, sex, duration, and severity of disease), the treatments have received, etc;
(4) intervention: type and/or form of Tai Chi, details of treatment and control, duration of treatment;
(5) outcome: outcomes measures, main data of the outcomes, time point of measurement, etc.

### 2.7 Methodological quality assessment

Studies quality will be assessed using the risk of bias tool provided by the Cochrane Handbook for Systematic Reviews of Interventions.^[43]^ This will assess the categories of bias for each study: selection bias (random sequence generation and allocation concealment), detection bias (blinding of outcome assessment), attrition bias (incomplete outcome data), reporting bias (selective reporting), and other bias (unbalanced base line, early termination). We will not report performance bias, considering the difficulties of blinding the participants and personnel in Tai Chi intervention studies. For each item, there are 3 potential bias judgements: “low risk,” “high risk,” or “unclear risk.” Trials that meet all criteria will be judged to have low risk of bias. A high risk of bias if at least 1 domain is assessed as at high risk of bias, and trials with insufficient information to judge will be classified as unclear risk of bias. Any disagreements will be resolved by discussion, with involvement of a third author (ND) where necessary.

### 2.8 Dealing with missing data

In the case of missing data included in study, we will contact the corresponding authors. If not successful, we will analyze available data to perform the outcome and assess the potential impact in the discussion.

### 2.9 Data synthesis

Data will be summarized by using risk ratios with 95% confidence interval (CI) for dichotomous outcomes or mean difference with 95% CI for continuous outcomes. It is anticipated that different scales may be used to report the same outcomes, in which case we will use the standardized mean difference (SMD). Statistical heterogeneity will be assessed using the *I*^2^ statistic (on the bases of characteristics of the included studies and the participants, details of the intervention or control, and types of outcome measurements). If the *I*^2^ statistic is <50% and the clinical heterogeneity among trials is acceptable, data will be pooled for statistical analyses using the Cochrane’ Review Manager software (V.5.3). Fixed effects model will be used to conduct the meta-analysis when the *I*^2^ statistic is <25%, otherwise random-effects model. When there is clinical heterogeneity or statistical heterogeneity (*I*^*2*^ > 50%), subgroup analysis or descriptive analysis will be conducted.

### 2.10 Subgroup analysis

Where data are available, subgroup analyses will be conducted to determine if effectiveness of Tai Chi is influenced by: different phrases of treatment, such as undergoing treatment, or in the post-treatment phase. Studies including patients receiving chemotherapy or radiotherapy as the initial cancer treatment or as treatment in the presence of metastasis or cancer recurrence will be classified as “undergoing cancer treatment stage,” while those studies including patients currently have gone through chemotherapy or radiotherapy will be defined as “post-cancer treatment stage.”

Also we will conduct subgroup analyses for time frame, intervention duration, cancer type, and so on if needed.

### 2.11 Sensitivity analysis

To ensure the robustness of evidence, we will perform sensitivity analysis to assess the impact of studies depending on study characteristics identified during the review process.

### 2.12 Publication bias

When sufficient RCTs are available for meta-analysis, we will conduct tests to explore publication bias using funnel plot asymmetry.

### 2.13 Ethics and dissemination

Ethical approval is not required because all data used in this study will be anonymous with no concerns regarding privacy. The findings will be disseminated through a peer-review publication, to inform both clinical practice and further research on Tai Chi and cancer.

## 3 Discussion

To the best of our knowledge, this is the first systematic review to identify the effect of Tai Chi practice on immunological function in cancer survivors based on major English and Chinese databases from a global perspective. This study includes a comprehensive search strategy across several health research-related databases to reduce the possibility of duplication and ensure the inclusion of representative studies.

The review will follow robust guidelines and the quality of the papers included will be assessed using a validated tool. Gaps in the literature will be identified to provide suggestion for future research on Tai Chi for cancer.

This review still has some limitations. We put more attention on immune measures level in cancer survivors, but less on the immunology-related clinical significance. There now exists an unprecedented opportunity for exercise-oncology researchers to unlock the potential therapeutic promise of exercise in cancer initiation and progression. But to do this require the design of research that involve multidisciplinary teams with expertise in exercise science, immunology, cancer biology, and clinical oncology.

## Acknowledgments

The authors are grateful for the valuable suggestions provided by Dr Li Bo (Beijing institute of traditional Chinese medicine, Beijing Chinese Medicine Hospital Affiliated to Capital Medical University).

## Author contributions

Xuejiao Wang and Lei Xu are joint first authors. Xuejiao Wang contributed to the conception of the study and wrote the draft of manuscript, and the final manuscript was revised by Lei Xu. The search strategy was developed by all of the authors. Qingyun He, Lei Xu, Xuejiao Wang, Ning Dai will search, extract data, assess the risk of bias, and complete the data synthesis. Ning Dai and Xingzhe Yang both contibuted to the methodology. Ruochong Wang and Libo Tan are responsible for project administration and supervision. Feng Li and Xuejiao Wang obtained funding and will arbitrate in case of disagreement and ensure the absence of errors. All authors have read and approved the final manuscript.
